# Quantitative proteomics identified 3 oxidative phosphorylation genes with clinical prognostic significance in gastric cancer

**DOI:** 10.1111/jcmm.15712

**Published:** 2020-08-05

**Authors:** Fei Su, Fen‐fang Zhou, Tao Zhang, Dan‐wen Wang, Da Zhao, Xiao‐ming Hou, Mao‐hui Feng

**Affiliations:** ^1^ Department of Oncology The First Hospital of Lanzhou University Lanzhou China; ^2^ Department of Biological Repositories Zhongnan Hospital of Wuhan University Wuhan China; ^3^ Center for Clinical Medicine of Peritoneal Cancer of Wuhan Wuhan China; ^4^ The Second Clinical Medical College of Lanzhou University Lanzhou China; ^5^ Department of Gastrointestinal Surgery Zhongnan Hospital of Wuhan University Wuhan China; ^6^ Clinical Cancer Study Center of Hubei Province Wuhan China; ^7^ Key Laboratory of Tumor Biological Behavior of Hubei Province Wuhan China

**Keywords:** biomarkers, DIA, gastric cancer, metabolic network

## Abstract

The aim of the present study was to explore the underlying mechanisms involved in gastric cancer (GC) formation using data‐independent acquisition (DIA) quantitative proteomics analysis. We identified the differences in protein expression and related functions involved in biological metabolic processes in GC. Totally, 745 differentially expressed proteins (DEPs) were found in GC tissues *vs*. gastric normal tissues. Despite enormous complexity in the details of the underlying regulatory network, we find that clusters of proteins from the DEPs were mainly involved in 38 pathways. All of the identified DEPs involved in oxidative phosphorylation were down‐regulated. Moreover, GC possesses significantly altered biological metabolic processes, such as NADH dehydrogenase complex assembly and tricarboxylic acid cycle, which is mostly consistent with that in KEGG analysis. Furthermore the higher expression of UQCRQ, NDUFB7 and UQCRC2 were positively correlated with a better prognosis, implicating these proteins may as novel candidate diagnostic and prognostic biomarkers.

## 1 INTRODUCTION

Globally, gastric cancer (GC) ranks the third among all types of malignancies in terms of cancer‐associated mortality.^1^ Despite the recent advances in diagnostics and therapeutics, the survival of patients with GC shows no significant improvement. GC is a multifaceted disorder and involves multiple factors, complicated biological processes and unpredictable outcomes.^2^ It is unreasonable that a single molecular marker could be accurately used for the prediction, prevention and personalized medicine (PPPM) practice in GC. Moreover, numerous molecular alterations at various levels, including DNA (genome), RNA (transcriptome), proteins (proteome) and metabolites (metabolome), have been demonstrated to participate in the progression of GC and are intertwined with diverse pathway networks. Recent studies have revealed that high‐throughput proteomics renders a promising approach to understand the progression of complex tumorigenesis and to identify potent cancer biomarkers.^2^ Proteomics is considered as a state‐of‐the‐art, large‐scale and systematic analytic approach, enabling the identification and quantification of proteins in specific biological specimens.^3^ Additionally, the difference between normal and lesion samples can be quantified using quantitative proteomics and this offers valuable evidence for identifying novel biomarkers and elucidating mechanisms underlying complicated biological processes.^4^ A comprehensive molecular characterization of gastric adenocarcinoma has been studied in 2014.^5^ Moreover, Mun et al^6^ and Ge et al^7^ reported the proteomic landscape of early‐onset and diffuse‐type GC, respectively. However, there are few studies of GC by using data‐independent acquisition mass spectrometry (DIA‐MS).

Data‐independent acquisition technology accurately quantifies proteins and is suitable specifically for biomarker research. To the best of our knowledge, the major objective of cellular biology is to understand the gene expression strategy under changing microenvironment in cancer development and progression. In the present study, we aimed to discover effective biomarkers of GC.

Over the past decade, many studies have elucidated the function of mitochondria in tumour cells, Zong et al^8^ which is suggestive that compromised mitochondrial bioenergetic function, along with changed phenotype, is a distinct feature of carcinogenesis.^9^ Moreover, the above outcomes have been validated in numerous malignancies in the past few years.^8^ Nevertheless, the property and origin of respiratory and metabolic alterations in GC cells remain unclear.

## 2 MATERIALS AND METHODS

### 2.1 Patients and tissue samples

For the proteomics and western blot analysis, 10 cases fresh frozen GC tissues and paired paracancerous tissues were obtained from The First Hospital of Lanzhou University, all the specimens were pathologically confirmed. Detailed clinical information of the patients is shown in Table 1. This study was approved by the Ethics Committee of The First Hospital of Lanzhou University.

**Table 1:** The clinical characteristics of the GC patients

Characteristics	Variable	Number
Age	<60	6
	≥60	4
Sex	Male	5
	Female	5
Tumour Size	<3 cm	4
	≥3 cm	6
Tumour differentiation	Well and moderately	3
	Poorly	7
Tumour infiltration	T1 + T2	4
	T3 + T4	6
Local lymph node metastasis	Negative	5
	Positive	5
Distant metastasis	M0	8
	M1	2

Abbreviations: GC, gastric cancer; M, metastasis; T, tumour.

### 2.2 Sample preparation

Samples were ground and lysed in the lysis buffer (8 mol/L Urea, 100 mmol/L Tris Hydrochloride, pH 8.0) supplemented with protease and phosphatase inhibitors (ThermoFisher Scientific) followed by 2 minutes of sonication (3 seconds on and 3 seconds off, amplitude 25%). Samples were centrifuged at 15 000 *g* for 10 minutes to collect total protein, followed by the assessment of protein concentration by Bradford protein assay (ThermoFisher Scientific).

### 2.3 Protein digestion

Protein (100 μg per sample) and 8 mol/L urea (100 μL) were transferred to a new Eppendorf tube, followed by the addition of 2 μL of 0.5 mol/L trichloroethylene (TCE) and incubated at 37°C for 1 hours; next, 4 μL of 1 mol/L iodoacetamide was added to the tubes and incubated at 24°C for an additional 40 minutes. Five volumes of pre‐chilled (−20°C) acetone were then added for protein precipitation overnight at −20°C. The next day, precipitates were rinsed twice with 1 mL pre‐chilled 90% acetone aqueous solution, followed by dissolution in 100 μL of 100 mmol/L tetraethylammonium bromide (TEAB). Sequence grade modified trypsin (Promega, Madison, WI) was added at a weight ratio of 1:50 (enzyme: protein) for protein digestion at 37°C overnight. Finally, the peptide mixture was desalted by C18 ZipTip and quantified using Pierce™ Quantitative Colorimetric Peptide Assay (# 23275).

### 2.4 Data‐dependent acquisition (DDA) mass spectrometry and database search

For the spectral library, the peptide mixture of 48 samples were re‐dissolved in 20 mmol/L ammonium formate in water (adjusted with ammonium hydroxide, pH 10.0), followed by fractionation using high pH separation by Ultimate 3000 system (ThermoFisher), connected to a reverse‐phase column (XBridge C18 column, 4.6 mm × 250 mm, 5 μm, Waters Corporation). Specifically, a linear gradient was utilized to conduct high pH separation, which started from 5% B buffer to 45% B buffer in 40 minutes (B: 20 mmol/L ammonium formate in 80% ACN, pH 10.0, adjusted with ammonium hydroxide). The column was subsequently re‐equilibrated under the initial condition for 15 minutes at a flow rate of 1 mL/min at 30°C. Next, ten fractions were collected, each of which was dried in a vacuum concentrator, followed by re‐dissolution in solvent A (A: 0.1% formic acid in water) and subsequent analysis was performed using online nanospray LC‐MS/MS on a Q Exactive HF X (ThermoFisher) connected to Waters nano ACQUITY UPLC system (Waters, MA, USA). Two microlitre peptide samples were loaded (analytical column, Acclaim PepMap C18, 75 μm × 25 cm) and separated with a 120‐minute gradient, from 3% to 30% B (B: 0.1% formic acid in ACN) at a flow rate of 400 nL/min. Moreover, the electrospray voltage of 2.1 kV versus the inlet of the mass spectrometer was employed. Raw data of DDA were processed and analysed using Spectronaut X (Biognosys, Schlieren, Switzerland) with default settings to generate an initial target list. Spectronaut X was established to search human UniProt protein sequence database (only reviewed entries, downloaded on February 1, 2019). Carbamidomethyl (C) was specified as fixed modification and Oxidation (M) as the variable modification. A Q value (FDR) cut‐off of 1% was applied on precursor and protein levels.

### 2.5 Quantification of proteins using DIA mass spectrometry

The mass spectrometer switched between MS and MS/MS state automatically under the DIA mode. The parameters were as follows: (a) MS: scan range (m/z) = 350‐1500; resolution = 60 000; AGC target = 4e5; maximum injection time = 50 ms; (b) HCD‐MS/MS: resolution = 30,000; AGC target = 1e6; collision energy = 32; stepped CE = 5%; (c) DIA was performed with variable isolation window, and each window overlapped 1 m/z, the window number was 45, and total cycle time was 3.98 seconds. Raw data of DIA were processed and analysed by Spectronaut X (Biognosys, Schlieren, Switzerland) with default settings. Retention time prediction type was set at dynamic iRT. Data were extracted by Spectronaut X based on the extensive mass calibration. The ideal extraction window was determined by Spectronaut X, which dynamically relied on iRT calibration and gradient stability. A Q value cut‐off of 1% was applied on precursor and protein levels. Decoy generation was set to mutate to apply a random number of AA position swamps (min = 2, max = length/2). All selected fragment ions passing the filters were used for quantification. The average top 3 filtered peptides, which passed the 1% Q value cut‐off, were used to calculate the major group quantities.

### 2.6 Validation of differentially expressed proteins (DEPs) by TCGA data sets

The prognostic value of key proteins was confirmed by Kaplan‐Meier (KM) survival analysis,^10^ and the gene expression levels in GC samples based on the clinical information from The Cancer Genome Atlas (TCGA) data sets were obtained using starBase^8^ (http://starbase.sysu.edu.cn/).

### 2.7 Bioinformatics analysis

R function prcomp from the stats package with default parameters was employed for principal component analysis (PCA) on every data set. Gene Ontology (GO) analysis was performed to explore the functional roles of DEPs in terms of biological processes, cellular components and molecular functions. Additionally, the Kyoto Encyclopedia of Genes and Genomes (KEGG) (http://www.genome.jp/kegg/) and Pathview (https://pathview.uncc.edu/) were used to explore the pathways related to DEPs. Online STRING database (https://string‐db.org/) was utilized to analyse protein‐protein interaction (PPI), followed by visualization using Cytoscape software (Version 3.7.0) (cut‐off value: 0.4). Scale‐free gene co‐expression networks were constructed by the WGCNA package. The Cytohubba was applied to identify hub genes and sub‐networks from complex interactome.^8^ Gene set enrichment analysis (GSEA) was conducted in GSEA v3.0 with all settings left default (minimum gene set size of 10 was excluded) along with the Molecular Signature Database (MSigDB) v7.0 collections: KEGG gene sets.^11^

### 2.8 Western blot

First, the tissues were sufficiently ground and total protein were extracted by RIPA buffer (Applygen) with a protease inhibitor cocktail and a phosphatase inhibitor cocktail and followed by western blot analysis.The following primary antibodies were used for western blot analysis: Anti‐UQCRQ antibody (ab241991; 1:1000), Anti‐NDUFB7 antibody (ab188575; 1:1500), Anti‐UQCRC2 antibody (ab203832; 1:800). GAPDH was used to be the control of cytoplasm proteins. The above antibodies were purchased from Abcam. After primary antibody detection, membranes were incubated with the appropriate goat anti‐rabbit or anti‐mouse secondary antibody (Abcam). All uncropped scans for blots were presented in corresponding Source Data file.

### 2.9 Statistical analysis

Unpaired Student's *t* test was employed to perform two‐group comparison. Welch's ANOVA Test was utilized to analyse the difference between the T and P groups. A *P*‐value < .05 as well as |fold change| >2 were used to filter DEPs.

## 3 RESULTS

### 3.1 DIA proteomics profiling and DEPs in GC

**Figure 1:**
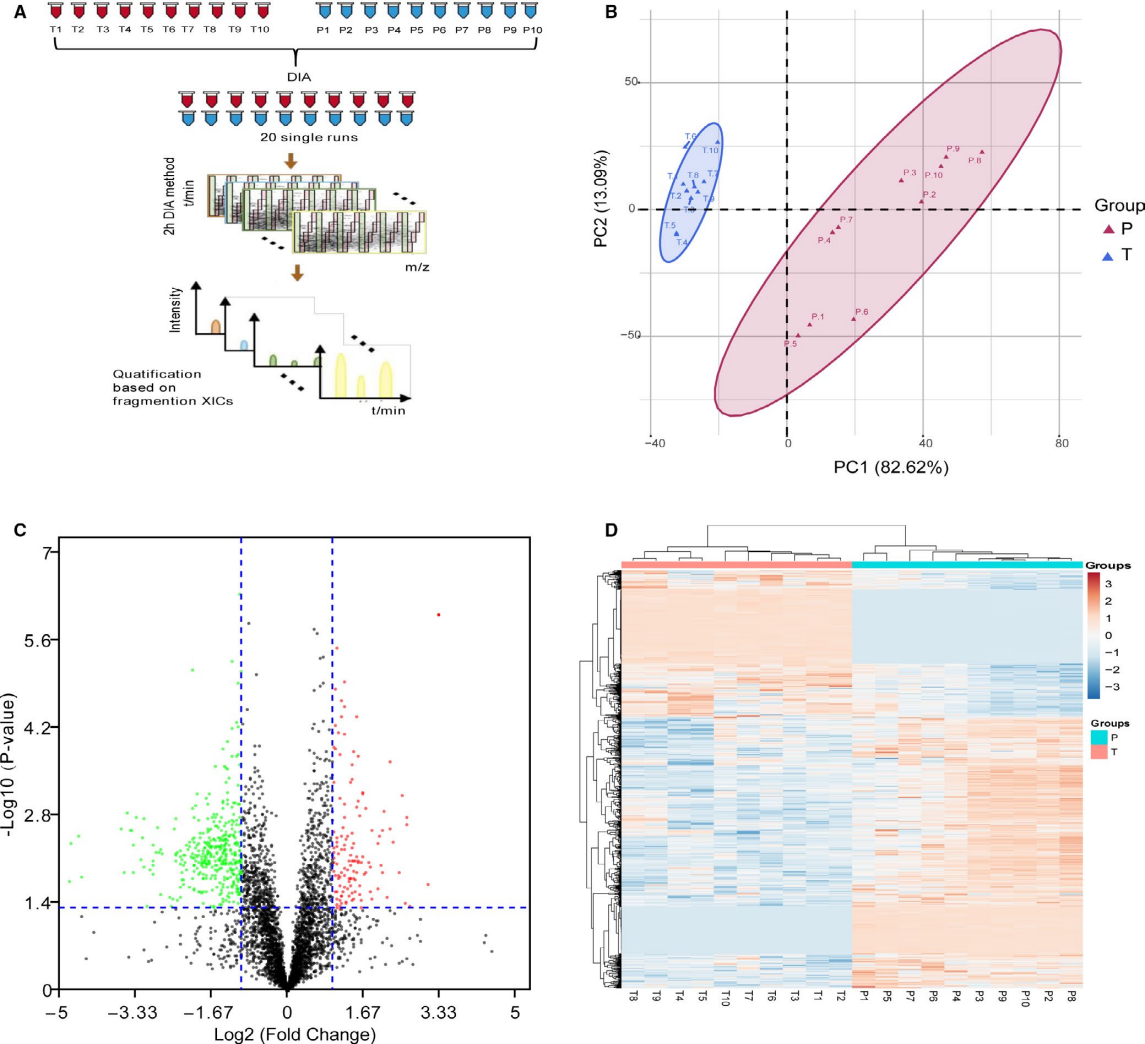
The DIA‐based quantitative proteomic landscape of the gastric cancer (T) and adjacent tissues (P). A, The simplified workflow of the DIA proteomics technology. B, Principal component analysis illustrating moderate clustering of gastric cancer and adjacent tissues. C, Volcano plot of log_2_ fold‐changes in DIA intensities (T versus P) reveal a large number of differentially expressed proteins. Proteins significantly elevated in T or P are coloured in red and green, respectively. D, Heatmap of the 745 significantly dysregulated proteins between T and P groups. Light blue represents the down‐regulated protein and orange indicates the up‐regulated protein in the gastric cancer group

To identify the candidate biomarker of GC, we performed a proteome‐wide screening experiment. In this study, 20 samples, including ten GC tissues and ten adjacent non‐tumourous tissues, were selected and subjected to DIA proteome analysis (Figure 1A). DDA analysis was performed for establishing a spectral library. Unsupervised hierarchical clustering, along with PCA, was employed to compare the filtered list containing 4322 proteins between GC and adjacent normal tissues. Most GC and adjacent samples clustered independently based on PCA (Figure 1B). Furthermore, 745 proteins were markedly changed (fold change > 2, *P*‐value < .05), of which, 485 and 260 proteins were down‐regulated and up‐regulated in the GC group, respectively (Figure 1C,D).

### 3.2 Functional classification of DEPs

**Figure 2:**
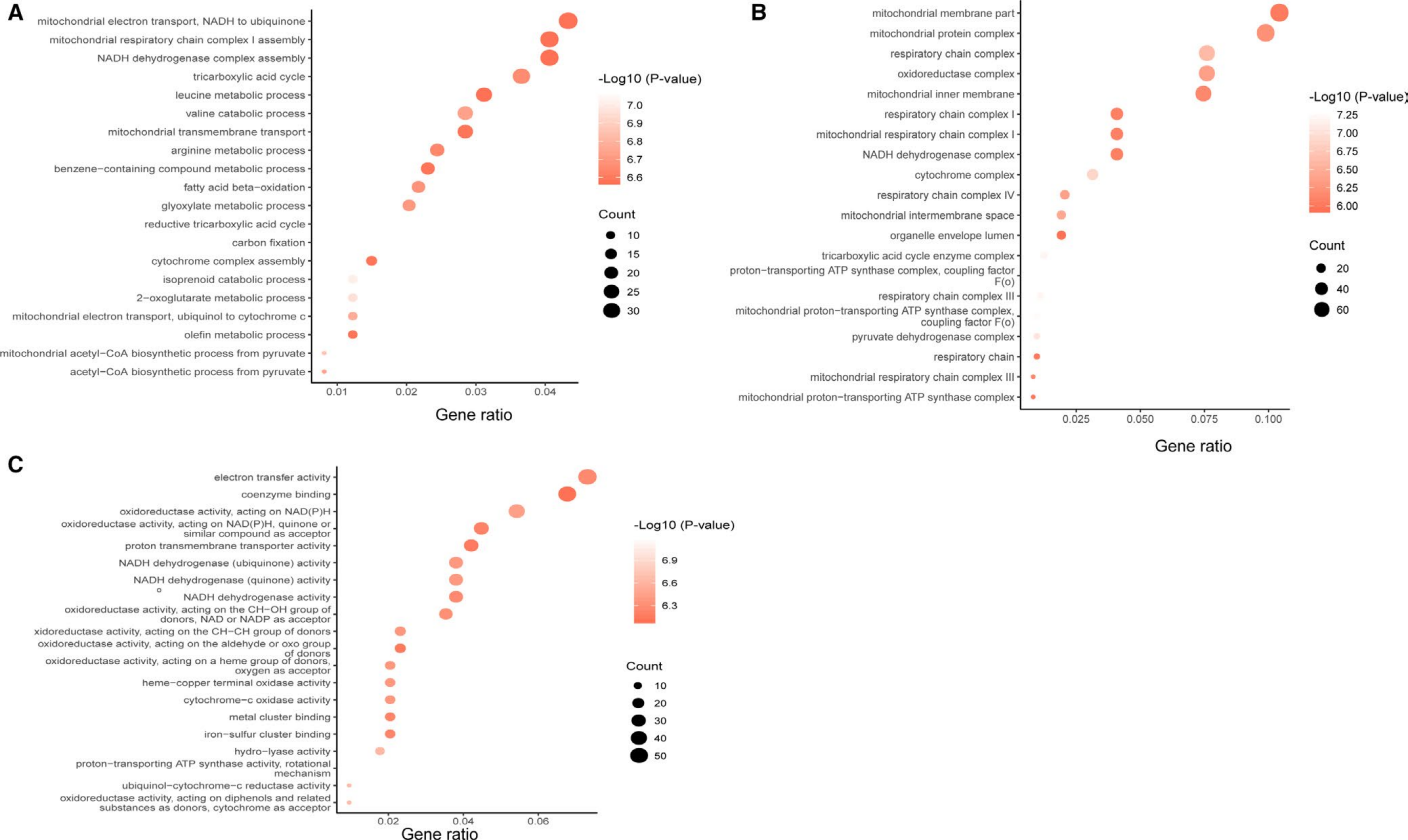
GO analysis of the differentially expressed proteins between the gastric cancer (T) and adjacent tissues (P). A, Gene Ontology analysis conducted for the differentially expressed proteins in terms of the top 20 ranking biological process. B, Gene Ontology analysis carried out for the differentially expressed proteins in terms of the top 20 ranking cellular component. C, Gene Ontology analysis performed for the differentially expressed proteins in terms of the top 20 ranking molecular function

All DEPs, including PLXDC2 and GPX8, were analysed using bioinformatics. GO enrichment analysis was performed for these DEPs in three aspects, including biological process (Figure 2A), cellular component (Figure 2B) and molecular function (Figure 2C). As shown in the bubble diagrams, relevant DEPs mostly altered biological metabolic processes, such as mitochondrial electron transport, mitochondrial respiratory chain complex I assembly, NADH dehydrogenase complex assembly and tricarboxylic acid (TCA) cycle (Figure 2A), and these processes were significantly altered in GC. Interestingly, these biological processes are mainly related to oxidative phosphorylation (OXPHOS). In terms of cellular component, majority of these DEPs are located in mitochondria, including the mitochondrial membrane, mitochondrial protein complex and respiratory chain complex (Figure 2B). The GC tissues are involved in electron transfer activity, coenzyme binding and oxidoreductase activity (Figure 2C).

### 3.3 Weighted gene co‐expression network analysis of GC proteome profiling

**Figure 3:**
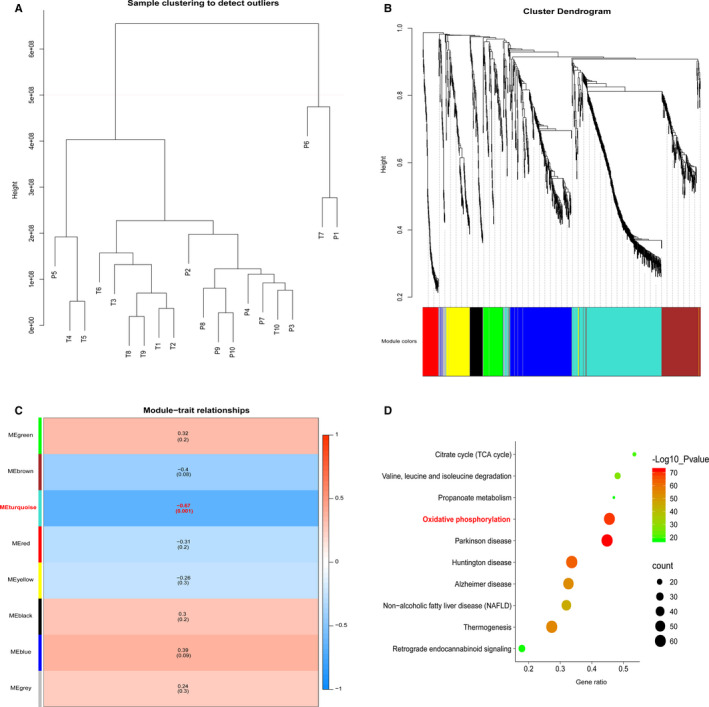
WGCNA analysis of the GC proteome profiling. A, Samples clustering were conducted to detect outliers between the gastric cancer (T) and adjacent tissues (P). B, Cluster dendrogram was generated by hierarchical clustering to show the modules of highly interconnected groups of genes between T and P groups. C, Heatmap was used to shown the correlation coefficient of module‐traits. D, KEGG pathway analysis of proteins in turquoise

To search the co‐expression modules of genes and the relationship between modules and pathogenesis of GC, WGCNA was applied on our proteome landscape of T and P through the R package WGCNA. Firstly, sample clustering was performed to detect variation and outliers of all 20 data sets. As shown, no outlier was identified and all of the samples were used for next step analysis (Figure 3A). Then the Pearson's correlation coefficients were calculated for genes in a pairwise manner, when threshold was set as 5, respectively. Using the matrix above, average linkage hierarchical clustering was performed on the 20 data sets to identify the densely interconnected gene modules (Figure 3B). As a result, one module labelled with turquoise was significantly co‐expressed (*P* = .001), and the correlation coefficient between tumour/normal was −0.67 (Figure 3C), which rendered an important function of genes for GC process in the module. To detect the fundamental function of the turquoise module, KEGG pathway analysis was used. As a result, genes in turquoise module were mainly related to oxidative phosphorylation, citrate cycle (TCA cycle) and so on (Figure 3D).

### 3.4 Pathway enrichment along with network analysis displayed relevant pathways of GC

**Figure 4:**
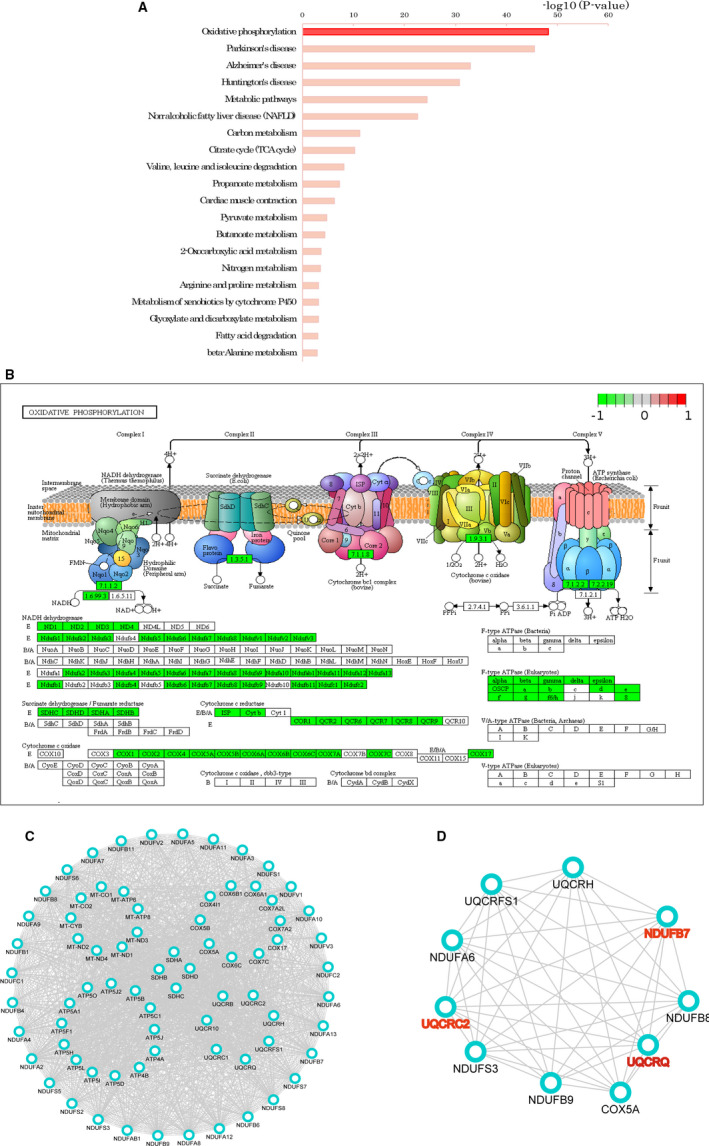
The pathway analysis of the differentially expressed proteins between the gastric cancer (T) and adjacent tissues (P). A, The significantly enriched top 20 ranking KEGG pathway of the DEPs. B, The oxidative phosphorylation signalling pathway (*P‐*value = 6.2e−49) played a crucial role in pathogenesis of GC. The green indicates the down‐regulated protein expression in the gastric cancer group. C, PPI network analysis of DEPs involved in oxidative phosphorylation by STRING database. D, The hub network of oxidative phosphorylation

KEGG database was searched for pathway enrichment analysis. By analysing DEPs, 38 pathways were significantly changed in GC tissues compared to those in adjacent non‐tumourous tissues. The significantly enriched top 20 ranking KEGG pathways of the DEPs are shown in Figure 4A. Coincidentally, all the identified DEPs involved in OXPHOS were down‐regulated. As shown in the pathway network, OXPHOS was significantly down‐regulated in GC tissues compared to that in adjacent tissues (Figure 4B). The interaction and correlation networks of the 76 DEPs involved in OXPHOS are shown in Figure 4C. For an in‐depth investigation into the DEPs in GC tissues, a hub subnetwork was constructed from the main network through the Cytohubba plugin (Figure 4D), which the TCGA database would use to verify the proteins for candidate markers.

### 3.5 Gene set enrichment analysis (GSEA) confirmed down‐regulation of oxidative phosphorylation in GC

**Figure 5:**
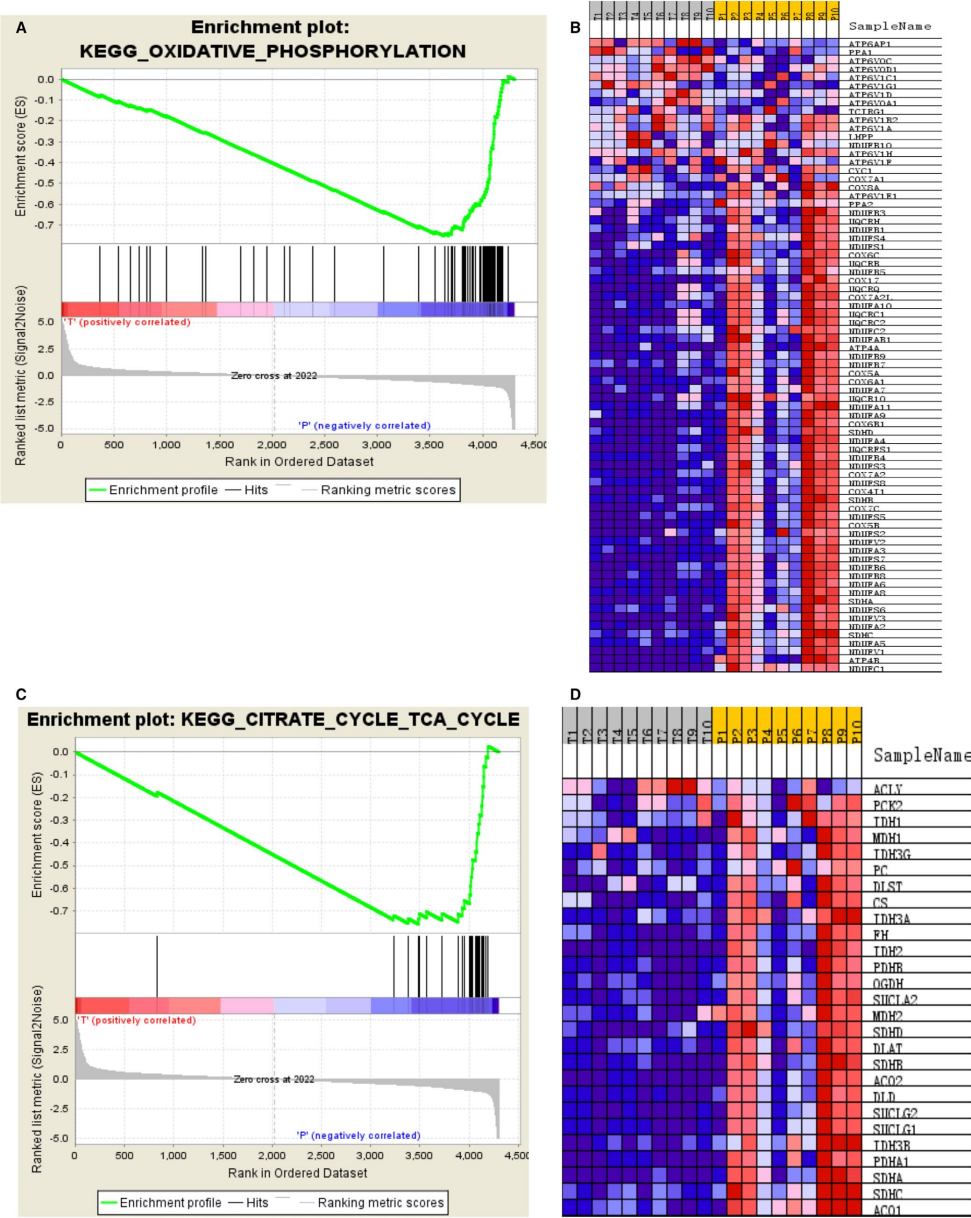
GSEA analysis of the whole quantified proteins between the GC and adjacent groups. A, GSEA comparing for enrichment of the oxidative phosphorylation pathway. NES = −2.73 and ***P < .001. B, Heat map of core enrichment genes in the gene set oxidative phosphorylation. C, GSEA comparing for enrichment of the oxidative phosphorylation pathway. NES = −2.22 and ***P < .001. D, Heat map of core enrichment genes in the gene set TCA cycle

Despite the crucial role of specific DEPs identification in an individualized patient for the possible biomarkers, it is equally important to elaborate the influenced protein pathways to approach pathological mechanisms, regardless of the possibility of insignificant results on a single member of such pathways according to the stringent filter criteria set for protein modulation.^12^ A GSEA‐based assessment of all detected proteins was conducted. However, the down‐regulated pathways in the GC were found to be mostly consistent with those in KEGG analysis, which included OXPHOS (Figure 5A) and TCA cycle (Figure 5C). The core enrichment genes in the OXPHOS and TCA cycle gene sets are shown in Figure 4B,D, respectively.

### 3.6 UQCRQ, NDUFB7 and UQCRC2 may be diagnostic biomarkers in GC

**Figure 6:**
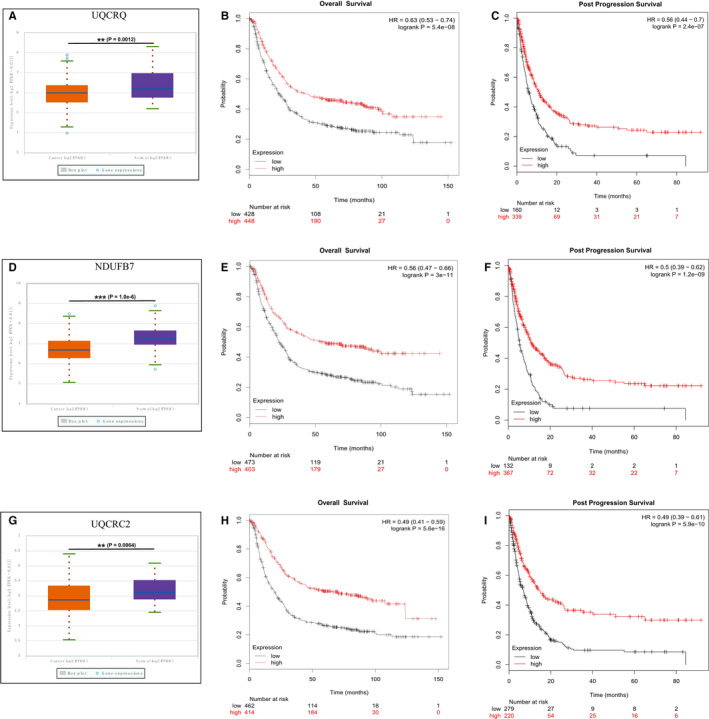
Gene expression and clinical significance in the TCGA. A, Box plots of UQCRQ mRNA expression (*P*‐value = .0012). B, C, Overall survival (OS) and Post Progression Survival (PPS) of 876 gastric cancer patients according to the UQCRQ status by Kaplan‐Meier Plotter database. D, Box plots of NDUFB7 mRNA expression (*P*‐value = 1.0e−6). E, F, Overall survival (OS) and Post Progression Survival (PPS) of 876 gastric cancer patients according to the NDUFB7 status by Kaplan‐Meier Plotter database. G, Box plots of UQCRC2 mRNA expression (*P*‐value = .0064). H, I, Overall survival (OS) and Post Progression Survival (PPS)of 876 gastric cancer patients according to the UQCRC2 status by Kaplan‐Meier Plotter database

**Figure 7:**
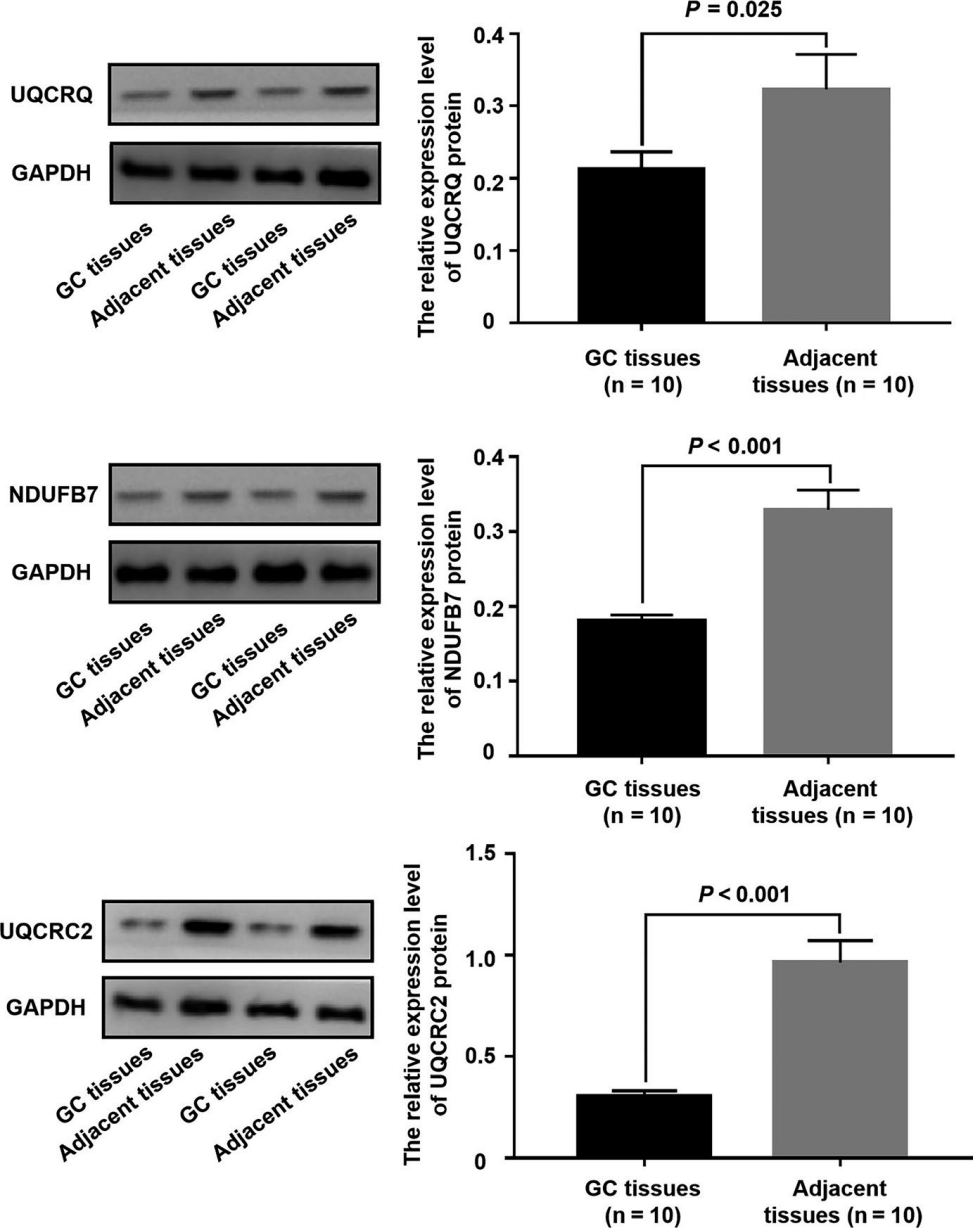
The expression level of UQCRQ, NDUFB7 and UQCRC2 protein in GC and adjacent tissues

Based on the hub subnetwork of OXPHOS (Figure 4D), we successfully retrieved the mRNA expression data of 10 DEPs from TCGA data sets using starBase, which contained 375 GC and 32 normal samples. The mRNA expression levels of three genes were significantly down‐regulated in GC, including UQCRQ (*P*‐value = .0012, Figure 6A), NDUFB7(*P*‐value = 1.0e−6, Figure 6B) and UQCRC2 (*P*‐value = .0064, Figure 6C). We also analysed the expression level of UQCRQ, NDUFB7 and UQCRC2 protein in the above 10 pairs of GC tissues and corresponding adjacent tissues by Western blot. Compared with adjacent tissues, the expression level of UQCRQ, NDUFB7 and UQCRC2 protein in GC tissues were significantly reduced (Figure 7).

Next, we assessed the prognostic value of these three proteins. The overall survival (OS) and post progression survival (PPS) of 876 gastric cancer patients according to the UQCRQ (OS HR = 0.63, 95% CI = 0.53‐0.74, *P* = 5.4e−08; PPS HR = 0.56, 95% CI = 0.44‐0.7, *P* = 2.4e−07), NDUFB7 (OS HR = 0.56, 95% CI = 0.47‐0.66, *P* = 3e−11; PPS HR = 0.5, 95% CI = 0.39‐0.62, *P* = 1.2e−09) and UQCRC2 (OS HR = 0.56, 95% CI = 0.47‐0.66, *P* = 3e−11; PPS HR = 0.5, 95% CI = 0.39‐0.62, *P* = 1.2e−09) status by Kaplan‐Meier Plotter database were shown in Figure 6. Obviously, there was a positive relationship between the high expression of UQCRQ, NDUFB7 and UQCRC2 with better prognosis, indicating that these genes may be used as new candidate diagnostic and prognostic biomarkers.

**Figure 8:**
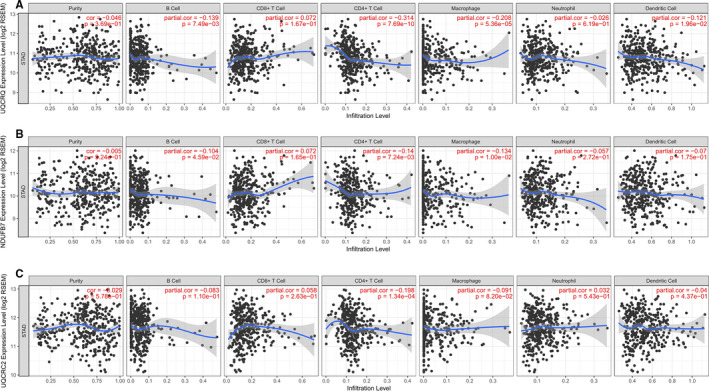
Correlation of UQCRQ, NDUFB7 and UQCRC2 expression with immune infiltration level in gastric cancer. The correlation of UQCRQ, NDUFB7 and UQCRC2 relative expression with tumour purity and gastric cancer infiltrating immune cells, like B cells, CD4+ T cells, CD8+ T cells, macrophages, neutrophils and dendritic cells were presented

Tumour microenvironment components, especially tumour infiltrating immune cells, such as B cells, CD+8 T cells, CD4+ T cells, macrophages, neutrophil and dendritic cells, playing a pivotal role in cancer occurrence and progression. To investigate the relationship between UQCRQ, NDUFB7, UQCRC2 and the diverse immune infiltrating cells, we focused on the correlations between the 3 genes and immune marker sets of various immune cells of GC in the TIMER and TCGA databases. As shown in Figure 8, UQCRQ, NDUFB7 and UQCRC2 expression showed significantly negative Pearson correlation with relative content of tumour infiltrating CD4+ T cells and macrophage cells, implying the role of the 3 genes not only in gastric cancer cells, but also in tumour infiltrating immune cells and tumour microenvironment. In sum, the results above suggested that UQCRQ, NDUFB7 and UQCRC2 were down‐regulated in GC samples, which may serve as candidate of prognostic biomarkers for GC patients.

## 4 DISCUSSION

GC ranks the fifth and third in morbidity and cancer‐associated mortality in all types of malignant tumours globally.^13^ Therefore, developing novel diagnostic and prognostic biomarkers of GC is essential. In recent years, quantitative proteomics analyses have been increasingly applied to provide new insights for the treatment of GC. Recently, the mitochondria have received considerable attention in cancer research. Mitochondria have been extensively investigated to address carcinogenesis due to their diverse and complicated functions, including biosynthetic metabolic modulation, cellular signalling and cell death, in addition to their role in energy production.^8^ Emerging evidence has revealed that altered protein and number of mitochondria,^8^ as well as aberrant mitochondrial components and function are related to multiple human malignancy progression and patient prognosis, suggesting the potential role of mitochondria in novel anti‐tumour therapeutic approaches by targeting metabolism or mitochondrial protein.^8^

In the present study, a comprehensive analysis of DEPs was conducted in GC tissues and adjacent tissues using DIA quantitative proteomics. Consequently, we successfully identified 260 down‐regulated and 485 up‐regulated proteins in the GC group. GO enrichment analysis showed significant changes in biological metabolic processes in GC. KEGG pathway and GSEA‐based analyses revealed that OXPHOS pathway and TCA cycle were significantly enriched. Our further validation analysis showed that NDUFB7, UQCRC2 and UQCRQ mRNA expression levels were significantly down‐regulated in GC; and the increased expression levels of UQCRQ, NDUFB7 and UQCRC2 were positively correlated with a better prognosis.

Warburg effect is one of the predominant features of rapidly growing tumour cells, which is capable of maintaining a high level of glycolysis to generate adequate ATP, regardless of the presence or absence of oxygen.^14^ OXPHOS is used for energy production in most cells and requires multiple respiratory enzymatic complexes, and the mitochondrial respiratory chain (consisting of four complexes in most organisms), located in the mitochondrial membrane. The electron transport is coupled with proton translocation from the mitochondrial matrix to the intermembrane space, and the generated proton gradient is utilized by ATP synthase for the catalysis of ATP generation following ADP phosphorylation.^15^

NDUFB7 gene is categorized into the hydrophobic group due to its NADH‐binding as well as oxidizing functions.^8^ NDUFB7 gene markedly affects PPARα response in oral tumorigenesis.^16^ Differential protein profiling demonstrated that Ndufb7 is down‐regulated through PPARα activation and response in oral tumorigenesis. In addition, we found that Ndufb7 is significantly down‐regulated in GC.

UQCRQ, a subunit of ubiquinol‐cytochrome c reductase complex III, part of the mitochondrial respiratory chain, encodes a ubiquinone‐binding protein of low molecular mass. In a previous study, mitochondrial dysfunction caused by UQCRQ defection has been reported to be involved in the pathogenesis of ulcerative colitis.^17^ UQCRC2 is a part of the ubiquinol‐cytochrome c reductase complex, also called complex III. UQCRC2, a key subunit of mitochondrial respiratory complex III, plays an important role in maintaining the structural and functional integrity of the mitochondria.^8^ Warburg first demonstrated that aerobic glycolysis was employed by tumour cells with rapid proliferation, which exerted an irreversible injury on OXPHOS.^18^ Additionally, excessive mitochondrial ROS generation could promote carcinogenesis and tumour progression.^8^

Mitochondrial dysfunction has been prevalently reported in tumour cells. Nevertheless, the notion that both mitochondrial metabolism and glycolysis are involved in tumour cells is controversial and widely challenged,^8^ and mitochondrial function plays a decisive role in the maintenance of cancer.^19^ Therefore, there have been various assessments and discussions over the changes in mitochondrial function and protein expression in different human malignancies.^8^ A homozygous missense mutation in UQCRC2 has been recently reported to possibly result in mitochondrial complex III deficiency, a relatively rare disease.^20^ Putignani et al have reported that UQCRC2 content in breast cancer cells is significantly lower than that in normal cells.^8^ Consistent with our outcomes on GC, Bai et al have discovered that UQCRC2 is down‐regulated in glioma tissues.^8^ Recently accumulative studies have elucidated the functions of mitochondria in the tumour, indicating the injured bioenergetic mitochondria as a hallmark of carcinogenesis.^8^ Moreover, mitochondrial alteration has been shown to be associated with cancer cell motility, invasion and chemo‐resistance.^8^ These results imply that UQCRQ, NDUFB7 and UQCRC2 might be involved in gastric carcinogenesis through different pathways. However, the correlation between UQCRQ, NDUFB7 and UQCRC2 in modulating mitochondrial energy generation and tumour progression remains unclear and needs to be confirmed by further experiments.

In conclusion, 485 down‐regulated proteins and 260 up‐regulated proteins were identified in the GC group. The GC tissues are mostly involved in electron transfer activity, coenzyme binding and oxidoreductase activity. Through a comprehensive analysis of DEPs, the mRNA expression of UQCRQ, NDUFB7 and UQCRC2 was found to be significantly down‐regulated in GC. However, the increased expression was positively related to a better prognosis, indicating that these proteins might be promising diagnostic and prognostic biomarkers for GC. Nonetheless, these results were obtained by bioinformatics analysis and require further validation.

## CONFLICT OF INTEREST

The authors declare no conflict of interest.

## AUTHOR CONTRIBUTIONS

**Fei Su:** Validation (equal); writing‐original draft (equal). **Fen‐fang Zhou:** Validation (equal); writing‐original draft (equal). **Tao Zhang:** Methodology (equal); visualization (equal). **Dan‐wen Wang:** Methodology (equal); visualization (equal). **Da Zhao:** Funding acquisition (equal); project administration (equal); resources (equal). **Xiao‐ming Hou:** Funding acquisition (equal); project administration (equal); resources (equal). **Mao‐hui Feng:** Methodology (equal); project administration (equal); resources (equal).

## CONSENT

All the patients signed the informed consent.

## Data Availability

The data used in this study are available from the corresponding authors upon request.
